# Th17 and Cognitive Impairment: Possible Mechanisms of Action

**DOI:** 10.3389/fnana.2019.00095

**Published:** 2019-11-19

**Authors:** Virginia Cipollini, Josef Anrather, Francesco Orzi, Costantino Iadecola

**Affiliations:** ^1^Sant’ Andrea Hospital, Sapienza University of Rome, Rome, Italy; ^2^Feil Family Brain & Mind Research Institute, Weill Cornell Medicine, New York, NY, United States

**Keywords:** Th17 cells, IL-17 cytokine, cognitive impairment, neuroinflammation, immune system, central nervous system

## Abstract

T helper 17 (Th17) cells represent a distinct population of immune cells, important in the defense of the organism against extracellular infectious agents. Because of their cytokine profile and ability to recruit other immune cell types, they are highly pro-inflammatory and are involved in the induction of several autoimmune disorders. Recent studies show that Th17 cells and their signature cytokine IL-17 have also a role in a wide variety of neurological diseases. This review article will briefly summarize the evidence linking Th17 cells to brain diseases associated with cognitive impairment, including multiple sclerosis (MS), ischemic brain injury and Alzheimer’s disease (AD). We will also investigate the mechanisms by which these cells enter the brain and induce brain damage, including direct effects of IL-17 on brain cells and indirect effects mediated through disruption of the blood-brain barrier (BBB), neurovascular dysfunction and gut-brain axis. Finally, therapeutic prospects targeting Th17 cells and IL-17 will be discussed.

## Introduction

It is well documented that T helper 17 (Th17) cells are important in the defense of the organism against opportunistic pathogens of fungal or bacterial origin, making them a fundamental part of the adaptive immune system. Th17 cells have also a role in barriers protection, especially in the bowel, where they regulate the host response to microbiota and maintain the intestinal homeostasis (Stockinger and Omenetti, 2017). It has also become clear that these cells are involved in the pathogenic mechanism behind several inflammatory disorders, such as multiple sclerosis (MS), inflammatory bowel disease (IBD), rheumatoid arthritis (RA) and psoriasis. However, the mechanisms by which Th17 cells, and their signature cytokine IL-17, exert their pathogenic effect remains poorly understood, especially in the central nervous system (CNS). Recent evidence provided insight into the trafficking of Th17 cells in and out of the brain and their potential contribution to brain diseases associated with cognitive impairment (Reboldi et al., 2009; Glatigny et al., 2011; Rothhammer et al., 2011; Engelhardt and Ransohoff, 2012). In this brief review article, we will analyze the available scientific evidence linking Th17 cells to brain diseases associated with cognitive impairment. We will also examine the mechanisms by which these cells enter the brain and induce brain damage. Finally, we will discuss the implications of targeting Th17 cells and IL-17 for new therapies for brain diseases.

## Th17 Cells Population

The innate immune system, after encountering specific pathogens, produces characteristic cytokines that are able to initiate the differentiation of naive CD4+ T cells into effector T helper cells, involving their T cell receptor (TCR) and other costimulatory molecules (Korn et al., 2009). The analysis of cytokine production, effector functions and transcription factors expression allowed identification of different subsets of CD4+ Th cells. In 1986, Mosmann and Coffman discovered two subsets of activated CD4+ T cells: Th1 and Th2 cells (Mosmann et al., 1986). Th1 cells differentiate in the presence of IL-12 and interferon-gamma (IFN-γ) and produce the very same cytokines in addition to tumor necrosis factor-α (TNF-α) and IL-2. Th1 cells are important in immune responses against intracellular pathogens, in resistance to mycobacterial infections and participate in the induction of several autoimmune diseases (Zhu and Paul, 2008; Wan, 2010). In contrast, IL-4 promotes the differentiation of Th2 cells. Th2 cells are essential in the defense against extracellular pathogens, in antibody class switching of B cells and are involved in promoting allergic diseases (Zhu and Paul, 2008; Wan, 2010).

More recently, other subsets of Th cells, which exhibit various effector functions, have been identified. Among them, Th17 cells represent a distinct population which differentiates in presence of IL-6 and transforming growth factor-β (TGF-β; Bettelli et al., 2006). Moreover, IL-21, together with TGF-β, influences Th17 cell differentiation and amplification of the response. Thus, IL-21 produced by differentiating Th17 cells can generate an autocrine loop of self-amplification, determining the expression of IL-23 receptor (IL-23R; Zhou et al., 2007). In turn, Th17 phenotype reinforces and increases its pathogenicity thanks to IL-23 signaling, which enhances the expression of its receptor (IL-23R; Wu et al., 2013). The transcription factor retinoic acid receptor-related orphan receptor gamma-T (RORγt) is expressed characteristically on Th17 cells. RORγt operates with different transcription factors, including RORα and signal transducer and activator of transcription 3 (STAT3; Miossec et al., 2009). Upon antigen-specific stimulation, Th17 cells generate a distinctive group of cytokines, including IL-17, IL-21, IL-22, IL-23, IL-6, IFN-γ and granulocyte monocyte-colony stimulating factor (GM-CSF), all of which participate in generating an inflammatory response (Annunziato et al., 2007; Miossec et al., 2009).

The function of Th17 cells is to activate immune responses against multiple pathogens, which cannot be efficiently managed by the immune responses of Th1 and Th2 cells. Thus, Th17 cells are able to engage and stimulate other immune cells, especially neutrophils and macrophages, and play a protective role against intracellular (Zhang et al., 2009; Zhou et al., 2009) and extracellular bacteria (Kudva et al., 2011), fungi (Conti et al., 2009) and other parasite (Miyazaki et al., 2010) infections. Th17 cells play a critical role on mucosal surfaces, such as lung and gut, where they promote the activation of pro-inflammatory danger signals that regulate the recruitment of neutrophils and the expression of anti-microbial factors. The induction of innate immune genes including neutrophil-activating factors, antimicrobial peptides and acute phase proteins allows Th17 cells to execute their defencing functions (Conti et al., 2009). However, because of their cytokine profile and ability to recruit other immune cell types, they are extremely pro-inflammatory and are implicated in the induction of several autoimmune diseases (Gaffen, 2009). Finally, in the small intestine lamina propria there is a population of T cells at steady state expressing RORγt; the presence of specific luminal commensal microbiota is necessary for their differentiation and accumulation (Shaw et al., 2012).

## IL-17

IL-17 is the main cytokine produced by Th17 cells (Park et al., 2005). The IL-17 cytokine family consists of six members (IL-17 A-F), the major isoform being IL-17A, which is secreted as disulfide-linked homodimer (Iwakura et al., 2011). IL-17A and IL-17F are likely to have similar biological activities and their signaling occurs through a common receptor, IL-17 receptor (IL-17R), composed of the subunit IL-17RA and IL-17RC. IL-17A, the most studied cytokine of this family, is a pro-inflammatory cytokine critical in the defense against microbial infections; it is involved in different conditions associated with systemic inflammation, including autoimmune diseases, metabolic disorders and malignancy (Gu et al., 2013).

The IL-17R family is composed of five members (IL-17RA to IL-17RE), homodimers or heterodimers (Walliser and Göbel, 2018). IL-17R is ubiquitously distributed and expressed on a wide type of cells, such as epithelial, endothelial cells and fibroblasts, as well as macrophages and dendritic cells. IL-17R contain certain conserved structural motifs including an extracellular fibronectin III-like domain and a cytoplasmic SEFIR [SEF (similar expression to FGF receptor)/IL-17R] domain, which allow IL-17R to engage the nuclear factor-kappa B (NF-κB) activator 1 (ACT1) adaptor and to induce NF-κB, mitogen-activated protein kinases (MAPKs) and CCAAT/enhancer-binding protein (C/EBP) pathways (Gaffen, 2009).

IL-17 promotes the expression of various cytokines through the activation of NF-κB (Witowski et al., 2004; Table 1). The activation of NF-κB requires tumor necrosis factor receptor-associated factor-6 (TRAF-6) adapter protein as a signal transducer and other major signaling intermediates include ACT1 and TRAF6-dependent TGF-β-activated kinase 1 (TAK1; Gu et al., 2013). Three different classes of MAPKs regulate IL-17 signaling: extracellular signal-regulated kinases (ERK1 and ERK2), stress-induced c-Jun N-terminal kinases (JNK-1 and JNK-2), and p38 MAPK. In addition, IL-17 can activate other downstream pathways including Janus kinases (JAKs) and activators of transcription (STATs; Witowski et al., 2004).

**Table 1:** Target genes of IL-17.

IL-17 target genes	
Proinflammatory cytokines	IL-6 (Witowski et al., 2004; Korn et al., 2009; Miossec et al., 2009; Iwakura et al., 2011; Nguyen et al., 2013)
	IL-1 (Witowski et al., 2004; Korn et al., 2009; Miossec et al., 2009; Iwakura et al., 2011)
	TNF-α (Witowski et al., 2004; Korn et al., 2009; Miossec et al., 2009; Iwakura et al., 2011)
	Granulocyte-macrophage colony-stimulating factor (GM-CSF) Granulocyte colony-stimulating factor (G-CSF; Witowski et al., 2004; Korn et al., 2009; Iwakura et al., 2011; Nguyen et al., 2013; Zenaro et al., 2017)
	Intercellular cell adhesion molecule 1 (ICAM-1; Witowski et al., 2004; Iwakura et al., 2011; Nguyen et al., 2013)
	Nitric oxide synthase 2 (iNOS; Iwakura et al., 2011)
	Cyclooxygenase-2 (COX-2; Witowski et al., 2004; Iwakura et al., 2011)
Chemokines and immune cell chemoattractants	CXCL1 (Korn et al., 2009; Iwakura et al., 2011; Nguyen et al., 2013; Zenaro et al., 2017)
	CXCL2 (Nguyen et al., 2013; Zenaro et al., 2017)
	CXCL5 (Nguyen et al., 2013)
	CXCL9 (Khader et al., 2007; Nguyen et al., 2013)
	CXCL10 (Khader et al., 2007; Korn et al., 2009; Nguyen et al., 2013)
	CCL2 (monocyte chemoattractant protein 1, MCP-1; Witowski et al., 2004; Park et al., 2005; Iwakura et al., 2011; Nguyen et al., 2013)
	CCL7 (Park et al., 2005; Nguyen et al., 2013)
	CCL20 (Park et al., 2005; Korn et al., 2009; Miossec et al., 2009; Nguyen et al., 2013; Zenaro et al., 2017)
Antimicrobial peptides	Defensins (Iwakura et al., 2011)
	Mucins (Chen et al., 2003)
	S100 proteins (Iwakura et al., 2011)
Matrix metalloproteinases	MMP1 (Iwakura et al., 2011)
	MMP3 (Park et al., 2005; Iwakura et al., 2011)
	MMP13 (Park et al., 2005; Iwakura et al., 2011)
Transcription factors	NF-κB (Witowski et al., 2004)
	C/EBPβ (Ruddy et al., 2004)
	C/EBPδ (Ruddy et al., 2004)

During the inflammatory response, IL-17 signaling is capable to improve cytokines and chemokines production, to increase and spread the pro-inflammatory response and to recall immune cells at the site of infection. To this end, IL-17 promotes the expression of genes encoding pro-inflammatory and hematopoietic cytokines, chemokines and immune cell chemoattractants, antimicrobial peptides and matrix metalloproteinases (MPOs) from fibroblasts, endothelial cells and epithelial cells (Table 1; Iwakura et al., 2008; Zenaro et al., 2017). These mechanisms guarantee the chemotaxis of inflammatory cells in response to inflammation (Iwakura et al., 2011; Nguyen et al., 2013).

IL-17A and other IL-17 family cytokines are not only produced by Th17 cells but also by other immune and non-immune cells. Indeed, under different conditions involving the activation of the immune system, other selective cell subtypes, such as macrophages, dendritic cells, natural killer cells and γδ T cells produce IL-17 (Korn et al., 2009). Moreover, intestinal Paneth cells can produce IL-17A (Takahashi et al., 2008).

Dysregulated IL-17 production and signaling have been involved in different autoimmune diseases (Gaffen et al., 2014), but the mechanisms are not yet fully understood. Recently, several pieces of evidence have revealed that blocking the activity of IL-12 and IL-23, IL-1, or IL-6, which are critical for Th17 cell differentiation and propagation, is effective for the treatment of inflammatory diseases, such as MS, IBDs, RA and psoriasis. Consistently, anti-IL-17A therapies are efficacious in these diseases, but the very same therapies increase the risk for opportunistic infections (Yamada, 2010).

## Th17 and IL-17 in Central Nervous System: Roles in Neurological Diseases

### Th17 and IL-17 in Multiple Sclerosis

Evidence from clinical studies and animal model studies, mostly experimental autoimmune encephalomyelitis (EAE), have revealed the role of Th17 cells in MS (Traugott et al., 1985). Both in MS and in EAE, T-lymphocytes are present in the brain parenchyma during the acute phase. Microarray-based approaches have demonstrated increased expression of IL-17 in MS plaques compared to normal, even before the identification of Th17 cells (Lock et al., 2002). The amount of Th17 cells is elevated also in cerebrospinal fluid (CSF) and peripheral blood of MS patients, especially during relapses (Brucklacher-Waldert et al., 2009; Durelli et al., 2009). Consistently with the increased cell number, the concentration of cytokine IL-17 increases with increased disease activity, as demonstrated by magnetic resonance imaging (MRI; Hedegaard et al., 2008). Further support to the link between Th17 cells, IL-17 and MS relapses comes from the observation that human Th17 cells are able to cross the blood-brain barrier (BBB) in MS lesions, enhancing neuroinflammation (Kebir et al., 2007).

Th1 cells are significantly involved in EAE and the production of INFγ and TNF-α is considered a marker for the ability of such cells to induce the disease. However, many studies have demonstrated how IL-23 cytokine, which is essential for Th17 cell population expansion, promotes EAE more robustly than IL-12 cytokine and INFγ-producing Th1 cells (Langrish et al., 2005; Touil et al., 2006). Passive transfer studies in mice indicate that Th17 cells promote EAE and their number correlates with disease severity. Treatment with anti-IL-17 antibody partially reverses the progress of EAE, attenuating the induction of pathogenic cytokines (Langrish et al., 2005). Different timing profiles of Th1 and Th17 cells in EAE has been demonstrated in a study by Murphy et al. (2010): 7 days post-immunization the amount of Th17 cells in spinal cord is major and lower at day 10; on the other hand Th1 cells are inferior at day 10 and increase at day 14. Indeed, evidence suggests that there is a faster clinical presentation of the disease when making an adoptive transfer of *ex vivo* differentiated Th17 cells, compared to adoptive transfer of *ex vivo* differentiated Th1 cells (Rothhammer et al., 2011). Furthermore, EAE is attenuated in IL-17 knockout mice (Komiyama et al., 2006), and mice deficient for RORγt, the key transcription factor for Th17 differentiation, presented a delayed onset and a mild progression of EAE (Ivanov et al., 2006). Lately, scientific interest has turned to the response of CNS-resident cells as targets of IL-17 signals. Both astrocytes and microglia express IL-17RA, but the role of IL17 signaling in these cells in MS and EAE needs to be investigated further (Waisman et al., 2015). It is also true that many studies have demonstrated that IL-17 has an important, but non-essential, function in EAE, considering the absence of resistance to disease after their deactivation (Haak et al., 2009; Rostami and Ciric, 2013) and that mice deficient in Th17 characteristic cytokines, such as IL-17A, IL-17F, IL-21 and IL-22, are particularly at risk of developing EAE (McGeachy et al., 2007). However, among Th17 cytokines, GM-CSF has an interesting encephalitogenic profile and a critical role during the effector phase of EAE. GM-CSF expression on T cells is regulated by IL-23 and the transcription factor RORγt and it sustained neuroinflammation, acting by myeloid cell infiltration. Unlike other cytokines, GM-CSF has a nonredundant role in promoting EAE and its secretion is able alone to render MOG-specific T cells autoaggressive and pathogenic (Codarri et al., 2011).

### Th17 and IL-17 in Ischemic Brain Injury

The relevance of the cytokines IL-17A and IL-17F as effector molecules responsible for neuronal damage in cerebral ischemia is still being discussed (Siffrin et al., 2010). Different studies indicate that IL-17 is involved in the delayed phase of the post-ischemic inflammatory cascade (1–5 days after onset of symptoms; Kostulas et al., 1999; Li et al., 2005; Haak et al., 2009; Shichita et al., 2009; Sutton et al., 2009; Erbel et al., 2011; Gelderblom et al., 2012; Hu et al., 2014; Siniscalchi et al., 2014; Benakis et al., 2016; Lv et al., 2016; Arunachalam et al., 2017; Zhang et al., 2017; Dolati et al., 2018). For instance, IL17-expressing cells are increased in the peripheral blood of post-ischemic stroke patients (Kostulas et al., 1999). Moreover, 3–5 days after stroke, IL-17-producing cells and IL-17A–positive lymphocytes are present in the brain parenchyma (Li et al., 2005; Sutton et al., 2009; Gelderblom et al., 2012), probably expression of a disparity between IL-17A-producing cells and regulatory T cells (Hu et al., 2014). A recent study demonstrates a marked decrease of peripheral Treg and a dramatic increment of Th17 cells, accompanied by the increase of IL-17A and RORγt expression, in patients at 1, 5 and 10 days after ischemic stroke (Dolati et al., 2018). Furthermore, IL-17 may contribute to atherosclerosis and plaque instability, a known risk factor for embolic stroke (Erbel et al., 2011).

Experimental studies also propose that IL-17 has a function in post-ischemic inflammation (Zhang et al., 2017). In an ischemic stroke model, IL-17A-producing γδ T cells were thought to enlarge infarct size, and both IL-17A and its receptor are increased after ischemic brain injury (Haak et al., 2009; Shichita et al., 2009). Moreover, γδ T cell trafficking from the gut to the meninges, which is modulated by the gut microbiota, may enhance ischemic neuroinflammation by secreting IL-17 and leading to chemokines production in the brain parenchyma, which, in turn, promotes the infiltration of the brain by monocytes and neutrophils (Benakis et al., 2016). A recent study (Arunachalam et al., 2017) demonstrated that brain-infiltrating γδ T cells expressing chemokine receptor CCR6 are a source of IL-17, inducing CXC chemokines production and neutrophils infiltration. In addition to immune system cells, CNS-resident cells can produce IL-17 during the progression of ischemic damage in the brain. Indeed, *in vitro* studies have shown that astrocytes can promote IL-17 production in response to pro-inflammatory stimuli (Meeuwsen et al., 2003). However, additional studies are required to better define the cellular origin of IL-17 during ischemic brain injury.

### Th17 and IL-17 in Alzheimer’s Disease

Alzheimer’s disease (AD) is the most common cause of cognitive impairment in the elderly. AD classical pathological hallmarks are intracellular neurofibrillary tangles and extracellular amyloid-β (Aβ) plaques. It is however well established that cerebrovascular alterations coexist in determining the development of the disease (de la Torre, 2017; Iadecola, 2017).

Recent studies have stressed neuroinflammation as a relevant mechanism in AD pathogenesis (Heppner et al., 2015; Marsh et al., 2016; Kisler et al., 2017). Highly insoluble Aβ fibrils and neurofibrillary tangles stimulate microglial activation, and consequently the production of proinflammatory cytokines and chemokines, and accumulation of inflammatory cells into the CNS. Aβ increases the production of reactive nitrogen intermediates, including nitric oxide (NO) and reactive oxygen species (ROS), by microglial cells, determining oxidative stress (Wang et al., 2015). Thus, subsequent oxidative stress promotes the increase of Th17/IL-17 axis. Both human and animal studies on AD have shown circulating leukocyte subtypes, such as lymphocytes, monocytes and neutrophils, in the brain parenchyma (Zenaro et al., 2015). However, whereas blood monocytes may contribute to Aβ clearance (Michaud et al., 2013), the importance of diverse leukocytes in AD pathology remains unclear.

Recently, it was suggested that Aβ acts a part in the chemotaxis and in the recruitment of neutrophils in the brains of mice overexpressing human mutant amyloid precursor protein (APP). The effect could be mediated by promoting the transition of lymphocyte function associated antigen 1 (LFA-1) integrin from the low- to the high-affinity binding state, thus enhancing neutrophil adhesion to the cerebral endothelium (Zenaro et al., 2015). The influx of neutrophils in the brain determines the production of IL-17, which is able to amplify the recall of neutrophils in the CNS, due to its harmful effect on neurons and BBB (Zenaro et al., 2015). Blockade of LFA-1 integrin or neutrophils depletion ameliorates memory deficits in AD animal models, revealing that neutrophils can promote cognitive dysfunction (Zenaro et al., 2015). Using a triple transgenic mouse model to replicate Aβ and tau neuropathologies, an increased activation of T and B lymphocytes was demonstrated. This observation may reflect the implication of the adaptive immune system in AD pathology. Moreover, cytokines quantification revealed high levels of IL-2, TNF-α, IL-17, and GM-CSF, suggesting a Th17 polarization (St-Amour et al., 2019). A different study on APP-overexpressing rats demonstrated a notable increase of IL-17, IL-22 and RORγt in the hippocampus, CSF and serum (Zhang et al., 2013). Finally, a recent study evidenced that the administration of antibodies anti IL-17 and the subsequent neutralization of IL-17 cytokine ameliorate cognitive impairment and amyloid-β-induced neuroinflammation in adult mice, as suggested by reduced Aβ1–42, glial fibrillary acidic protein (GFAP), S100 proteins and MPOs (Cristiano et al., 2019). These effects support the synergic role of IL-17 and related cytokines in promoting AD neuroinflammation and neurodegeneration (Solleiro-Villavicencio and Rivas-Arancibia, 2018).

In human AD brains, both CD4+ and CD8+ T cells are detected in the parenchyma and vascular endothelium and their number is higher than in healthy controls (Town et al., 2005). Activated T cells in the brain, particularly Th1 or Th17 cells, tend to escalate the inflammatory cascade. In turn, the secretion of inflammatory cytokines such as IFNγ or IL-17 by T-cells could promote AD neuropathology (Browne et al., 2013; Zenaro et al., 2017). Furthermore, an elevation of IL-17 and IL-23 in the serum is observed in AD patients (Chen et al., 2014). The results obtained by Saresella et al. (2011) regarding naive lymphocytes from AD patients have demonstrated an increase of Th17 cytokines production, including IL-21, IL-6, and IL-23 and expression of Th17 transcription factor RORγt in AD patients compared to MCI subjects and healthy control subjects (Saresella et al., 2011). Oberstein et al have noticed an increase of Th17 cells in subjects with MCI due to AD pathology compared to subjects with MCI due to other pathologies and control subjects, and a relevant association between the level of Th17 cells and amyloidopathy, expressed by the decrease of the ratio of Aβ42/Aβ40 (Oberstein et al., 2018). Recently (Tahmasebinia and Pourgholaminejad, 2017), different studies in both human and animal models have proved the relevance of Th17 lymphocytes in early AD.

Finally, scientific evidence gives importance to choroid plexus dysfunction in AD pathogenesis. Indeed, it was shown that, with aging, the choroid plexus may have a negative impact on brain function (Baruch et al., 2013). Type I IFN, including IFN-α and IFN-β, is involved in reducing inflammation and its enhanced expression in aged choroid plexus may represent a physiological response to moderate neuroinflammation in aging. However, Baruch et al suggested that the IFN I response of the choroid plexus during normal aging negatively regulates adult neurogenesis and spatial learning and memory and its persistent expression becomes counterproductive (Baruch et al., 2014). It has also been suggested that the amyloid burden causes dysfunction of choroid plexus epithelium through oxidative stress and inflammatory signaling. The resulting remodeling of tight junction (TJ) leads to barrier dysregulation and increased cellular influx (Bergen et al., 2015).

## How Do Th17 Cells Enter The CNS?

There are different routes and trafficking signals for leukocytes to enter the CNS. The venular route is the main one and allows the migration through the walls of post-capillary venules from the microvessels to the parenchymal perivascular space. A second route is through the walls of the meningeal vessels into the subarachnoid space. Finally, migration *via* choroid plexus let leukocytes access CSF from blood. During inflammation, leukocytes prefer to enter the CNS through the venular and the meningeal routes, on the other hand the choroid plexus route is involved in CNS immunosurveillance under both physiological and pathological conditions (Vajkoczy et al., 2001; Kerfoot and Kubes, 2002; Ransohoff et al., 2003; Shechter et al., 2013; Zenaro et al., 2017). Recently, meningeal lymphatic vessels were discovered confirming the relationship between the peripheral immune system and CNS. Indeed, the meningeal lymphatic system contributes to drainage of CSF components and allows the entry of immune cells into cervical lymph nodes. These capabilities are guaranteed by meningeal lymphatic endothelial cells expressing a unique transcriptional signature and an appropriate migratory profile (Louveau et al., 2018). Meningeal lymphatics are involved in immunosurveillance and neuroinflammation and their ablation ameliorates EAE pathology, opening a promising target for therapeutic intervention (Louveau et al., 2018).

### Th17 and Choroid Plexus Gateway

The choroid plexus is composed of blood vessels and connective tissue, surrounded by a specialized epithelial monolayer creating the blood-CSF barrier and producing the CSF. The plexus acts as a neuro-immune connection and allows the exchange of signals between the brain and peripheral circulation. Recently it was evidenced that the choroid plexus regulates the entry of leucocytes to the non-inflamed CNS in different conditions such as physiological immunosurveillance and early phases of EAE (Ransohoff, 2009; Reboldi et al., 2009). Kunis et al. (2013) have recognized IFN-γ as a key regulator of leucocyte trafficking. This pathway allows leukocytes to enter the SNC through increased IFN-γ receptor expression on choroid plexus and intensified IFN-γ production by immune cells and it is enhanced during inflammatory conditions by the synergic action of TNF-α. IFN-γ signaling upregulates intercellular cell adhesion molecule 1 (ICAM-1) expression on the apical side of choroid plexus endothelium, promoting the transmigration of T cells subtypes, and induces CXCL9 and CXCL10 chemokines, which attract T cells by binding to CXCR3 receptors (Kunis et al., 2013).

Considering the link between Th17 cells and EAE (Reboldi et al., 2009; Kunis et al., 2013) investigated whether IL-17 can act on the choroid plexus, facilitating the trafficking of macromolecules. Their finding suggested that IL-17, but not IFN-γ, upregulating chemokine CCL20 expression on choroid plexus epithelial cells, promotes the entry of CCR6+ cells and promotes the onset of EAE. As suggested by Sie et al. (2014), CCR6+ Th17 cells enter the subarachnoid space using the choroid plexus epithelium (which expresses the CCR6 ligand—CCL20) and initiate the process of neuroinflammation. Indeed, according to previous results (Reboldi et al., 2009), CCR6 is crucial for the entrance of Th17 cells in the first phase of EAE. CCR6 is particularly implicated in CNS leukocytes trafficking and is supposed to be a brain-specific determinant: it is expressed not only on Th17 cells but also on cells that produce both IL-17 and IFN-γ, on a subset of Th1 cells, on B cells and on T regulatory cells (Reboldi et al., 2009). Moreover, in EAE, CCR6 ligand-CCL20, which is normally expressed on choroid plexus epithelial cells, is also expressed on astrocytes. Indeed, during inflammation phase, activated astrocytes may recall leukocytes to the brain parenchyma (Reboldi et al., 2009). After entering the CSF through the choroid plexus, Th17 cells are distributed on the pial surface and the perivascular space (Virchow-Robin space). Here, resident antigen-presenting cells expose self-antigens that are identified by Th17 cells. Thus, the activation of Th17 cells enhances the production of cytokines and chemokines, inducing BBB dysregulation and promoting the influx of other inflammatory cells, including Th1 cells, neutrophils and activated monocytes (Reboldi et al., 2009). Therefore, once inflammation has been established, redundant mechanisms allow leukocytes to enter the CNS.

## How Does Th17 Cells Promote Brain Injury?

**Figure 1:**
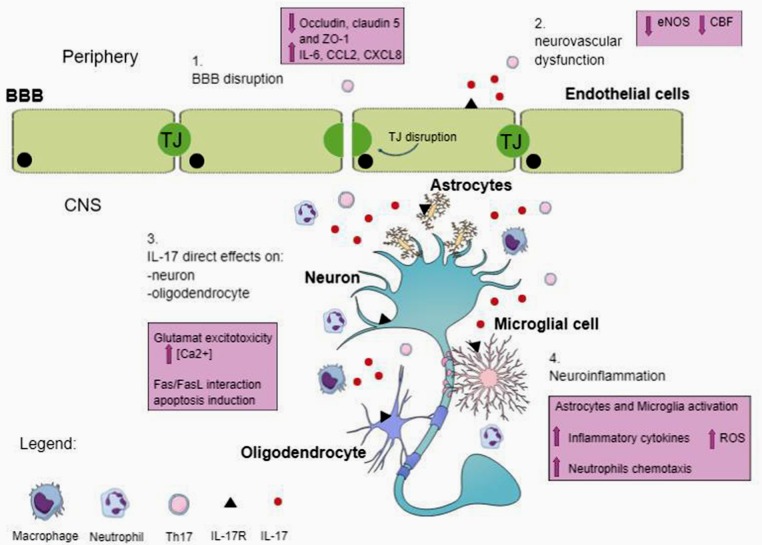
Possible mechanisms of action of T helper 17 (Th17) and IL-17 in central nervous system (CNS). IL-17 is produced by Th17 and other cells, including natural killer cells, and γδ T cells. IL-17 receptor (IL-17R) is located on different cell types in the CNS. Circulating IL-17 provokes blood–brain barrier (BBB) disruption by altering TJ and cell-adhesion molecule expression on endothelial cells. The breakdown of BBB favors the entrance in the CNS of peripheral immune cells, including Th17 cells, neutrophils and other immune cells. Moreover, IL-17 allows cerebral endothelial cells to stop producing endothelial NO (eNOS), resulting in reduced cerebral blood flow (CBF) and endothelial dysfunction. In the CNS, IL-17 has direct effects on neurons and oligodendrocytes, inducing damage. Additionally, IL-17 signaling on microglia and astrocytes determines the expression of inflammatory cytokine, amplifies the production of reactive oxygen species (ROS) and promotes neutrophils chemotaxis and accumulation (see text).

The mechanisms by which Th17 cells and their signature cytokines participate in the pathogenesis of neurological diseases have not been completely elucidated. Here, we will briefly review some of the potential mechanisms including direct effects of IL-17 on brain cells, and indirect effects mediated through disruption of the BBB or neurovascular dysfunction (Figure 1).

### Direct Effects

Several studies in EAE suggested a direct effect of IL-17 on CNS-resident cells (Vajkoczy et al., 2001). As mentioned before, ACT1 is essential for IL-17R signaling (Lee et al., 2015). The deletion of ACT1 in resident CNS cells reduces EAE severity, acting on oligodendrocyte progenitors and altering their response (Sie et al., 2014).

In addition, *in vitro* studies have revealed that IL-17 blocks the differentiation and reduces the survival of oligodendrocyte lineage cells (Kang et al., 2013). In a different study, IL-17 exacerbated oligodendrocyte loss by TNF-α activity and inhibited their progenitor cell differentiation (Paintlia et al., 2011).

Th17 cells have been suggested to interact directly with neurons, in the setting of EAE, forming antigen-independent immune synapse-like contacts. The adhesion molecule LFA1 is crucial in this immune synapse formation. These neuron–Th17 cell contacts result in increased intracellular [Ca2+], leading to Ca2+ overload and neuronal damage (Siffrin et al., 2010). Kebir et al. (2007) suggested that Th17 lymphocytes transmigrate efficiently across BBB endothelial cells and exhibit the cytolytic enzyme granzyme B, which showed the ability to destroy human fetal neuron-enriched cultures. Finally, a potential mechanism for Th17 pathogenic effect has been observed following hippocampal injection of Aβ1–42, where Th17 cells infiltrated the brain parenchyma after BBB disruption, leading to IL-17 and IL-22 elevations and neuroinflammation (Zhang et al., 2013). Therefore, Th17 cells might induce neuronal apoptosis by engaging the cell death receptor Fas through its interaction with the ligand FasL, expressed by Th17 cells, suggesting a direct injury to neurons by Th17 cells through the Fas/FasL pathway (Zhang et al., 2013).

### Th17 and BBB Disruption

The BBB isolates the CNS from the systemic circulation, and it is essential to sustain the optimal microenvironment in the CNS. The BBB is attributable to brain endothelial cells, connected by TJs preventing the paracellular passage of substances from blood to brain. They also have low levels of vesicles for macromolecular transport (transcytosis), compared to peripheral endothelial cells (Town et al., 2005; Abbott et al., 2010). Brain endothelial cells are endowed with specific transporters that regulate metabolites trafficking. They are characterized by the expression of different adhesion molecules, including intercellular and vascular adhesion molecules such as vascular cell adhesion molecule 1 (VCAM-1), and P- and E-selectin (Sweeney et al., 2009). In the normal BBB, mononuclear cells penetrate by a process of diapedesis directly through the cytoplasm of the endothelial cells, without TJs disruption (Abbott et al., 2010). Otherwise, during inflammatory processes, cytokines and other agents may open the TJs between endothelial cells and mononuclear cells may then penetrate by both transcellular and paracellular processes (Abbott et al., 2010). Recently, *in vivo* studies on EAE confirmed that caveolar transcytosis is important for T cell subtypes trafficking through the BBB/blood-spinal cord barrier (BSCB), however, Th17 cells migration acts with a caveolin-independent TJ remodeling occurring in early phase of EAE (Lutz et al., 2017). A study by Wimmer et al. (2019) has given new insight on Platelet endothelial cell adhesion molecule-1 (PECAM-1) in the mechanism of paracellular T cell diapedesis across the BBB. Indeed, in EAE, the presence of PECAM-1 on endothelial cells is required in regulating BBB permeability, influencing T-cells trafficking and promoting paracellular over transcellular T-cells diapedesis (Wimmer et al., 2019).

Previous studies have demonstrated that BBB disruption and lymphocytes activation are responsible for T-cells entry into the CNS (Farkas et al., 2003). In both human brain endothelial cells (Kebir et al., 2007; Rahman et al., 2018) and murine brain endothelial cell line (Huppert et al., 2010), IL-17 was shown to disrupt barrier integrity. It is also known that human brain endothelium exhibit IL-17 and IL-22 receptors, which activated increased BBB permeability (Kebir et al., 2007). Both *in vitro* and *in vivo* studies revealed that Th17 cells can modify and alter BBB TJs. IL-17A, IFN-γ and zonulin can increase BBB and small intestinal epithelial barrier permeability *in vitro*, remodeling TJs protein expression (ZO-1, claudin-5, and occludin) and underlying actin cytoskeleton (Rahman et al., 2018). After IL-17 and IL-22 cytokines induction, BBB endothelial cells may secrete CCL2 (or MCP-1), promoting transmigration of CD4+ lymphocytes (Kebir et al., 2007). A study by Wojkowska et al. (2017) suggested that the selective direct action of IL-17 on endothelial cells determines the release of CCL2 and CXCL1 chemokines, which is dose-dependent (Wojkowska et al., 2017). Moreover, IL-17 promotes IL-6 and CXCL8 expression by brain endothelial cells. The resulting opening of the BBB facilitates CNS inflammation, recalling additional CD4+ lymphocytes (Kebir et al., 2007). The mechanism by which IL-17 and IL-22 alter the BBB are not completely understood. A recent study (Huppert et al., 2010) found that IL-17 can disrupt the BBB promoting superoxidate production by NAD(P)H oxidase and xanthine oxidase. Excessive oxidative stress determines dysregulation of TJs and of TJs protein expression and activation of the endothelial cytoskeleton resulting in impaired barrier function. In rats with hippocampal injection of Aβ, Th17 cells penetrate the brain parenchyma from the disrupted BBB, as evidenced by increased expression of RORγt, a specific Th17 transcription factor, and by RORγt-immunoreactive cell localization around the damaged blood vessels (Baruch et al., 2014). A study on relapsing-remitting MS (RRMS) patients showed that CSF IL-17A levels are increased and correlated with BBB damage in RRMS, as demonstrated by CSF/serum albumin quotient (Qalb; Setiadi et al., 2019).

### Th17 and Neurovascular Dysfunction

The endothelial cells of BBB cooperate with neurons, astrocytes, interneurons, microglia and pericytes, shaping the so-called Neurovascular Unit (NVU), which is essential for the brain’s functional and structural integrity. All these components of NVU interact in response to physiological stimuli and assure adequate brain perfusion through different mechanisms (McConnell et al., 2017). Cerebral endothelial cells release vasoactive agents, regulating vascular tone. Indeed, cerebrovascular autoregulation maintains cerebral blood flow (CBF) stable during variations in arterial pressure within a certain range (Kisler et al., 2017). In addition, neural activity increases CBF, a phenomenon known as functional hyperemia or neurovascular coupling (Iadecola, 2017). The mechanisms of functional hyperemia involve the release of vasoactive signals from multiple brain and vascular cells, resulting in a highly coordinated vascular response involving the entire cerebrovascular tree, but resulting in a focused increase in flow restricted to the activated area (Iadecola, 2017).

Previous studies suggest that hypertension can disrupt the neurovascular coupling and the endothelium vasomotor function, promoting neurovascular dysfunction (Faraco et al., 2016). Hypertension and neurovascular dysfunction are influenced by both innate and adaptive immune responses (McMaster et al., 2015). In support of this hypothesis, there is an increase in the levels of IL-17 in hypertension and autoimmune diseases associated with it (i.e., pre-eclampsia, systemic lupus erythematosus; Madhur et al., 2010; Nguyen et al., 2013). Angiotensin II-induced hypertension model allows detecting increased T cells infiltrating the adventitia and periadvential fat of vessels (Guzik et al., 2007). Moreover, other studies showed an increase in Th17 cells and production of IL-17 and an increase in the mobilization of T lymphocytes and macrophages that cross the vascular wall (Madhur et al., 2010). In angiotensin II-induced hypertension model, TNF-α plays a critical role and synergize with IL-17 to modulate inflammatory responses, resulting in an increase of vascular resistance which promotes hypertension and end-organ damage. Indeed, IL-17 can alter vascular reactivity, promoting a proinflammatory milieu in the vessel wall and increasing superoxide production. Finally, IL-17, activates other pro-inflammatory, such as IL-6 and IL-1β, involved in neurovascular dysfunction and hypertension (Nguyen et al., 2013).

IL-17R is found on endothelial cells. Subsequent activation of IL17R determines RhoA/Rho-kinase-mediated endothelial NO synthase (eNOS) Thr495 inhibitory phosphorylation (Nguyen et al., 2013). Considering their function in determining an endothelial dysfunction and then developing hypertension, inhibition of RhoA/Rho-kinase may be useful in these disorders when associated with increased IL-17 (Nguyen et al., 2013).

In hypertension models, a distinctive role in the neurovascular and neurocognitive dysfunction is assigned to brain-resident perivascular macrophages (PVMs), a population of innate immune cells (Faraco et al., 2016). They act by activating angiotensin type 1 receptors, responsible for superoxide-producing enzyme NAD(P)H oxidase induction and subsequent ROS production. Producing vascular ROS, PVMs promote neurovascular dysfunction. Furthermore, the increase of BBB permeability by inflammatory cytokines enhances these effects. In AD models, PVMs are found in the perivascular space stimulating toxic vascular oxidative stress. This contributes to Aβ-induced neurovascular dysfunction (Park et al., 2017).

Finally, animal models have demonstrated that gut microbiota may favor angiotensin II-induced hypertension and neurovascular dysfunction. The absence of gut microbiota protects from these events, inhibiting the trafficking of inflammatory myelomonocytic cells in the vasculature, which is at least partly mediated by IL-17 pathway (Karbach et al., 2016).

### Th17 Response and Gut-Brain Axis

The gut microbiota is composed of all the microorganisms, which colonized the gastrointestinal tract. Host physiology, particularly the immune system regulation, is modulated by these intestinal microbes (Fung et al., 2017). In the last years, many studies investigated the association between the gut and the brain, however the different mechanisms behind this relationship are still partly unknown.

The microbiota affects the immune system in different ways; immunomodulation is particularly involved in microbiota-gut-brain communication.

Recently, special attention has been paid to mucosal tissues and their local immune compartments, i.e., mucosa-associated lymphoid tissues (MALT), and to their influence on mediated T-cells immunity.

The gut microbiota influences the activities of astrocytes and microglia, which in turn act on various neuropsychological processes such as neuronal development, neurotransmission, BBB integrity and CNS immune system activation (Fung et al., 2017). Moreover, the microbiota modulates peripheral immune responses, determining relevant effects on brain functions (Cryan and Dinan, 2012; Fung et al., 2017).

Braniste et al. (2014) have proved that germ-free mice, which lack all gut microbiota, show lower expression of TJ proteins claudin 5 and occludin and increased BBB permeability. It was shown that the short-chain fatty acid butyrate, a major metabolite of Clostridia species, was sufficient to revert BBB dysfunction in germ-free mice. Although the mechanisms underlying the beneficial effects of short-chain fatty acids on BBB function remain unclear, identifying how the gut microbiota is able to influence the permeability of BBB, could help to find new mechanisms by which to restore a weak BBB in diseases.

Th17 cell polarization can be influenced by several factors including environmental factors. Studies on environmental influence permit to find an interesting link between gut response and IL-17 increase in periphery, in particular situation such as a high salt diet (Kleinewietfeld et al., 2013).

Indeed, high-salt conditions promote Th17 polarization by the activation of the p38/MAPK pathway including nuclear factor of activated T cells 5 (NFAT5) and serum/glucocorticoid-regulated kinase 1 (SGK1; Kleinewietfeld et al., 2013). Previous works have shown that SGK1 regulates Na+ transport and cell salt (NaCl) homeostasis. Moreover, *in vitro* and *in vivo* studies have revealed that even a modest elevation of salt concentration can encourage the expression of SGK1, the upregulation of IL-23R and the differentiation of Th17 cell (Wu et al., 2013).

Under high-salt conditions, it is possible to observe a highly pathogenic Th17 cell population, as demonstrated by the increased production of pro-inflammatory cytokines (i.e., GM-CSF, TNF-α and IL-2; Kleinewietfeld et al., 2013). Moreover, Kleinewietfeld et al. (2013) observed that high salt diet determines a critical worsening of EAE in mice, coinciding with an increase of Th17 cells.

High salt diet leads to cerebral endothelial dysfunction in mice causing cognitive impairment (Faraco et al., 2018). This was associated with increased Th17 polarization in the small intestine, without evidence of cerebrovascular inflammation. Dietary sodium determines a Th17 response in the gut with an increase in the IL-17 plasma level. IL-17, with its vasotoxic effects, allows cerebral endothelial cells to stop producing eNOS, involving the Rho kinase (ROCK)-dependent inhibitory phosphorylation of eNOS and resulting in reduced CBF and cerebrovascular dysfunction (Faraco et al., 2018). In addition, endothelium-dependent vasorelaxation was impaired in mice fed with high salt diet, indicating that in this condition reduced CBF and impaired vascular reactivity might account for resulting neuronal dysfunction and cognitive disorder (Faraco et al., 2018).

There is also evidence that sodium, besides its direct effects on T cell polarization, regulates Th17 cells by altering the composition of intestinal microbiota. High salt diet depleted lactobacilli and monocolonization of germ-free mice with *L.murinus* was sufficient to suppress salt-induced Th17 differentiation, improved EAE pathology, and ameliorated salt-dependent hypertension (Wilck et al., 2017).

## Therapeutic Considerations

The recent identification of IL-17 secreting T cells as crucial contributors of the tissue damage in several neurological diseases raises the possibility to use Th17 cell–IL-17 pathway as a target for therapies in diseases associated with Th17 polarization. From animal model studies, we know that treatment with monoclonal antibodies anti-IL-17 greatly reduces EAE severity (Langrish et al., 2005). Furthermore, in a murine stroke model, the neutralization of IL-17A results in reduced neutrophil infiltration, decreased infarct size and improved neurologic outcome (Gelderblom et al., 2012).

In several autoimmune diseases, human clinical trials with humanized neutralizing IL-17A antibodies have yielded encouraging results (Genovese et al., 2010; Hueber et al., 2010). Recently, a clinical trial with Secukinumab, an IL-17A-neutralizing antibody, provided the first evidence that IL-17A-blocking antibody can reduce MS activity, although the results showed only a positive trend in reducing relapse rates, improving MRI parameters but not clinical outcome measures (Havrdová et al., 2016).

Tissue inflammation is driven by different inflammatory cytokines (IL-17, IL-17F, IL-22, IL-26, and GM-CSF), whose production is influenced by the activity of Th17 cells. Therefore, targeting Th17 cells instead of individual effector cytokines would be a greater benefit. Some efforts have been made regarding targeting RORγt, *via* small molecule inverse agonists, and IL-23/IL-23R signaling, but more investigations are still needed (Xiao et al., 2014).

Moreover, as already mentioned, the gut-brain axis has shown great relevance in CNS functions and in cognitive impairment. Avoiding excessive salt intake may be a strategy to prevent the damage deriving from brain microvasculature impairment, which leads to altered brain function and cognitive impairment (Faraco et al., 2018). Finally, protecting the brain endothelium, a common target for all cardiovascular risk factors, may be a novel, unexplored therapeutic target to prevent neurovascular dysfunction, which is relevant in the pathogenesis of neurodegenerative diseases including AD (Iadecola, 2017).

In conclusion, emerging evidence point to a role of Th17-IL-17 in a wide variety of neurological diseases associated with cognitive impairment, ranging from neurovascular to neurodegenerative diseases. However, several arguments need to be discussed concerning mainly the mechanism of their pathogenic effect and their potential value as therapeutic targets. Considering the expanding role of adaptive and innate immunity in a growing number of cerebral and systemic pathologies, additional research in this field is required and may lead to new insights into the pathogenesis and treatment of these highly prevalent and devastating neurological disorders.

## Author Contributions

CI contributed substantially to the conception and design of this review and provided final approval of the version to publish. VC took the lead in writing the manuscript. CI, JA and FO provided critical feedback and helped shape the research, analysis and manuscript. All authors revised the manuscript critically for important intellectual content.

## Conflict of Interest

The authors declare that the research was conducted in the absence of any commercial or financial relationships that could be construed as a potential conflict of interest.
